# Some Observations on the Relative Efficacy of the Various Treatments of Pernicious Anæmia
*From a thesis presented for the degree of M.B. Cantab.


**Published:** 1932

**Authors:** G. L. Ormerod


### SOME OBSERVATIONS ON THE RELATIVE EFFICACY OF THE VARIOUS TREATMENTS OF PERNICIOUS ANÆMIA.*

BY

G. L. ORMEROD, M.A., M.B., B.Chir.

---

*Historical.*—Pernicious anæmia is a disease which has been known now for rather over a century. It was in 1822 that Dr. Combe, a Scottish physician, described a case which was undoubtedly one of pernicious anæmia, he being the first to differentiate between a special type of anæmia and the anæmias as a whole which had been known for ages past. From the autopsy he could find no cause for the condition, but thought that it was probably due to some disorder of the digestive system.

In 1849 Dr. Thomas Addison, of Guy's Hospital, described a disease like that reported by Combe, but laid more stress on there being no previous loss of blood from any cause whatever. He distinguished this anæmia from those due to some definite known cause by naming it idiopathic anæmia. It is this type of anæmia which now bears his name. He suggested that it might be caused by some form of fatty degeneration.

The next stage in the history of pernicious anæmia was in 1867, when Professor Biermer, of Zurich, described cases which were identical with those of Addison and Combe without knowing that they had

* From a thesis presented for the degree of M.B. Cantab.

previously been described. Biermer drew attention to the tendency towards capillary hæmorrhages, but so far no mention had been made of the important fact of remissions and relapses. As to the ætiology, Biermer's views differed from those of Addison on an important point, as he thought that the disease was simply a complication of symptoms, due to poor living and sepsis amongst other things. He also described the condition as occurring in cancer and *dibothriocephalus latus* infestation, and called the whole group of cases progressive pernicious anæmia, and included Addison's idiopathic anæmia as one of these. This view of the ætiology of the disease was taken up by the majority of the continental investigators, and there the condition is now commonly known as Biermer's anæmia.

Unfortunately, Addison's name in connection with the disease seems to have been forgotten, until in 1875 Dr. Pye-Smith, who was a pupil of Addison, pointed out that Addison was the first to describe the disease as a separate clinical entity, and this was acknowledged in 1878 by Professor Eichhorst when he wrote that "this disease was first described in England."

In the next twenty-five years William Hunter and Ehrlich were the chief contributors towards our knowledge of pernicious anæmia. In 1881 Ehrlich's discovery of the value of aniline dyes in staining blood films led to a great advance in the same cause.

Hunter realized that the disease involved not only the hæmatopoietic system, but also the alimentary and central nervous systems ; thus he confirmed the original view of Combe seventy-five years previously. Hunter also strongly attacked Biermer's view of grouping various anæmias of different ætiology under one heading, and firmly upheld Addison's view of its idiopathic nature. His chief work, though, was to do with sepsis of the whole alimentary tract, and he formed the opinion that the disease was due to a hæmolysis of the red-blood corpuscles in the portal system. He recognized, though, that this sepsis was not the actual cause of the disease, but that it paved the way for some hypothetical hæmolytic agent, the primary source of which he could not find.

Ehrlich, on the other hand, after the discovery by Pepper and Cohnheim in 1875 of the megaloblastic appearance of the bone marrow, stated that this condition was due to degeneration, and brought forward the view that this megaloblastic degeneration, as it was called, was the essential pathognomonic finding in pernicious anæmia. Later, Peabody investigated the bone marrow during life by puncture of the tibia, and discovered that the megaloblastic hyperplasia occurred during relapses, and during remissions the normoblasts were formed as usual.

In recent years the increased knowledge of the formation of the blood and of the reticulo-endothelial system, the discovery of the constant achlorhydria, and in particular Castle's work on the stomach, have all helped to shed further light on the ætiology of the disease.

*Diagnosis.*—The most common symptoms and signs in a case of pernicious anæmia are :—

(1) Anæmia with a relatively high hæmoglobin percentage, giving a colour index above 1·0.

(2) A blood film showing nucleated blood corpuscles, poikilocytosis, polychromasia with punctate basophilia and anisocytosis with the average size of the red cells increased to 8 or 9$\mu$, which gives the high colour index.

(3) A slight hæmolytic jaundice which, together with the anæmia, gives the lemon-coloured tint to the patient.

(4) Achlorhydria.

(5) Glossitis (first described by Barclay in 1851).

(6) Remissions and relapses.

(7) The response to modern treatment. It has been said by several investigators that no improvement following upon liver or pig's stomach therapy indicates that the patient is not suffering from true pernicious anæmia.

Of these, the most important for a diagnosis of the disease are an increase in the average size of the red cells, the presence of megaloblasts in the blood, and the absence of free HCl in the gastric juice.

During the last eleven years experiments have been performed the results of which have completely altered the outlook and prognosis of pernicious anæmia. In 1920 Whipple and Robins performed experiments on dogs. From these animals they removed half their quantity of blood and then tried the effect of various types of diet on the time taken for the blood to return to its normal quantity. They found that a diet liberal in protein gave the quickest return to normal, but that whether it was derived from liver, heart or skeletal muscle made little difference. Two years later Jenks confirmed their findings.

In 1925 Whipple and Robins reported further experiments with dogs. This time they bled them repeatedly and so kept up a continuous severe anæmia. They kept the hæmoglobin percentage to a constant of 40—50, and by measuring the total amount of hæmoglobin in the blood which had to be withdrawn each time to do this they determined the rate of blood regeneration. On trying the effect of various diets again they discovered that a diet rich in liver produced by far the most rapid hæmoglobin and red-blood corpuscle re-formation ; next to this came kidney, then heart muscle and fourthly skeletal muscle. With regard to iron, they found that this was inert in the short anæmias but useful in the prolonged ones. Arsenic they found sometimes inert and sometimes it produced an improvement. They conclude their article by stating : " Under a variety of experimental conditions the reaction to iron and arsenic may vary, yet the favourable reaction to liver feeding is a sustained and uniform maximum."

Following on the work of Whipple and Robins, Minot and Murphy tried the effect of liver on pernicious anæmia. They thought that liver had its success by stimulating the maturation of the megaloblasts for three reasons : (1) there occurs a marked increase in the reticulocytes ; (2) this increase is inversely proportional to the initial red-cell count, as there are few megaloblasts in the marrow when the count is relatively high than low ; (3) in secondary anæmia the marrow does not contain so many megaloblasts, and liver does not increase the reticulocytes. White, of Washington, thought that the Kupffer cells may be responsible for the stimulus, as they probably belong to the same mesoblastic origin as the endothelial cells themselves, which when injected into a rabbit produce an overgrowth of the mesoblastic cells.

Shortly after the effectiveness of whole liver in the treatment of pernicious anæmia was discovered, Cohn made studies on the effective part of the liver and isolated an effective fraction of it which represents about 1 per cent. of the whole liver. It contains nitrogen, but is non-protein in nature ; it is precipitated by alcohol and is soluble in water. West also obtained an effective part. This extract is now marketed by several firms, and can be obtained as a liquid or a powder.

The next and latest advancement in the treatment of pernicious anæmia is the employment of hog's stomach. During the last few years much attention has been drawn to the constant symptom of achlorhydria. Castle followed this up in 1927 by a series of experiments. First he gave beefsteak to a patient with pernicious anæmia with no beneficial results. Then he gave artificial gastric juice—made from hydrochloric acid and pepsin—and still no improvement followed. Next he tried beef pre-digested with artificial gastric juice, with the same result. Finally he gave beefsteak to a normal person and allowed it to digest in his stomach for an hour; he then withdrew the chyme and gave it to a patient with pernicious anæmia; immediate improvement followed in the same way as with liver. It is thought from this that some substance is formed by the digestion of beef protein in a healthy stomach which is necessary for blood formation, and that it is stored in the liver chiefly and to a certain extent in the kidneys.

These experiments were followed up by trying the effect of normal stomach tissue on patients suffering from pernicious anæmia. The hog's stomach was used as it more closely simulates that of man than does any other animal. Desiccated preparations of the stomach were soon evolved, and are put up in the form of a powder.

The following cases of pernicious anæmia have been treated at the Bristol Royal Infirmary or General Hospital, and have been seen by the writer during the whole or part of their treatment.

| Name. Age. Admitted. | Complaint. | History of present illness. | General condition. | Alimentary system. | C.N.S. | Blood. | Treatment. | Progress. |
|---|---|---|---|---|---|---|---|---|
| C.B. 58 years. 17.4.26. | Anæmia. | Loss of weight and strength since December last. No appetite. Vomits. Sleeps well. | Pale yellow colour, gums and lips very blanched. | Tongue is smooth, moist and furred. Teeth, dentures. Spleen, not felt. | Normal. | R.B.C. 1,100,000. Hb. 25 per cent. Poikilocytosis, anisocytosis, few nucleated reds. V.D.B. 1·5 units indirect. | Blood transfusion. Liq. Arsenicalis m.3. Ac. HCl dil. ʒi.q.q.h. Kaolin ʒi b.d. 1.5.26. Transfusion. | 2.5.26. R.B.C. 2,190,000. General condition better. 12.6.26. R.B.C. 2,900,000. Discharged and to continue treatment at home. 6.9.26. Readmitted very ill. Transfusion. 7.9.26. Dead. |
| A.B. 40 years. 16.4.26. | Anæmia and loss of appetite. | Been feeling ill for six months. Numbness in hands for three months. | Mucous membrane almost colourless. | Teeth, fair. Tongue, furred, smooth, shiny. Spleen, not felt. No free HCl. | Normal except for subjective reactions. | R.B.C. 1,400,000. Hb. 54 per cent. Size, 7·95$\mu$. Polychromasia, poikilocytosis, anisocytosis. Nucleated reds. | Liq. Arsenicalis m.3 and HCl. | 28.4.26. R.B.C. 1,900,000. 17.5.26. Still improving and to continue treatment at home. May, 1931. Been on liver extract for last three years and her present condition is good. |

| Name. Age. Admitted. | Complaint. | History of present illness. | General condition. | Alimentary system. | C.N.S. | Blood. | Treatment. | Progress. |
|---|---|---|---|---|---|---|---|---|
| A.J.<br>45 years.<br>18.1.27. | General weakness. | Started six months ago. Diarrhœa and pins and needles in legs for last three months. | Typical yellow colour. | Spleen, just felt.<br>No free HCl. | No change. | R.B.C. 1,250,000.<br>Hb. 30 per cent.<br>C.I. 1·3.<br>W.B.C. 6,000.<br>Poikilocytosis, anisocytosis. | Liq. Arsenicalis<br>HCl dil.<br>m.20 t.d.s. | 3.2.27.<br>R.B.C. 2,000,000.<br>Hb. 40 per cent.<br>C.I. 1·1.<br>17.2.27.<br>R.B.C. 2,300,000.<br>Hb. 40 per cent.<br>C.I. 0·95.<br>1.3.27.<br>R.B.C. 2,750,000.<br>7.3.27.<br>R.B.C. 3,100,000.<br>Hb. 45 per cent.<br>Still has tingling in legs, but feels much better. Discharged to continue treatment.<br>Died a few weeks later |
| S.P.<br>48 years.<br>18.5.27. | General weakness.<br>Cough. | Has been getting short of breath for last year.<br>Very sore tongue for last month. | Pale colour.<br>Dulness and crepitations at left apex. | Superficial glossitis.<br>Spleen felt.<br>No free HCl. | No change. | R.B.C. 2,800,000.<br>Hb. 55 per cent.<br>C.I. 1·1.<br>W.B.C. 9,000.<br>Poikilocytosis.<br>Nucleated reds. | Liq. Arsenicalis m. 3 t.d.s. increased gradually to m. 10 t.d.s.<br>10.6.27.<br>Injections of Arsenious Acid gr. 1/20 twice a week.<br>6.6.27.<br>Transfusion.<br>20.6.27.<br>Transfusion. | 1.6.27.<br>R.B.C. 2,400,000.<br>General condition worse.<br>20.6.27.<br>R.B.C. 2,500,000.<br>1.7.27.<br>R.B.C. 1,700,000.<br>6.6.27.<br>General condition very bad.<br>10.6.27.<br>Improved.<br>20.6.27.<br>Worse again.<br>R.B.C. 1,200,000.<br>Hb. 20 per cent.<br>22.6.27.<br>Died.<br>P.M., signs of pernicious anæmia and cavities in both lung apices. |

| | | | | | | | | |
|---|---|---|---|---|---|---|---|---|
| *B.S.*<br>*34 years.*<br>*28.5.27.* | *Anæmia and weakness.* | *Started three months ago losing weight. Insomnia.* | *Very thin and anæmic.* | *Spleen, not felt. No free HCl.* | *Vibration sense in legs poor. Legs spastic. Plantar reflex.* | *R.B.C. 1,600,000. Hb. 45 per cent. C.I. 1·5. Average diam. 7·9μ (4μ to 11μ). W.B.C. 5,310.* Poikilocytosis, polychromasia, punctate basophilia. V.D.B. reaction 2·5 units indirect. | *Arsenious acid injections, HCl.* | *5.6.27.*<br>*R.B.C. 1,900,000.*<br>*27.6.27.*<br>R.B.C. 2,260,0000.<br>13.7.27.<br>R.B.C. 3,670,000.<br>Hb. 70 per cent.<br>Discharged on same treatment.<br>April, 1931.<br>Has been on liver extract and HC1 for last two years and feels very well.<br>R.B.C. 4,150,000.<br>Slight variation in size and shape. |
| M.G.<br>47 years.<br>18.4.27. | Feeling tired. | Has had several attacks of anæmia which improved on taking Blaud's pills ; has been worse the last few months. Tongue sore for six months. | Sallow complexion. | Teeth good. Tongue red and raw. Spleen, slightly enlarged. No free HCl. | Normal. | R.B.C. 2,500,000. Hb. 55 per cent. C.I. 1·1. Great variation in size and shape. Few nucleated red cells. | Liq. Arsenicalis m. 3 t.d.s. up to m. 10 t.d.s., HCl dil.<br>28th June. Raw liver, but could not take it. Then cooked liver.<br>Feb., 1928. Taking 1 lb. cooked liver a day and HCl.<br>April, 1931. ½-lb. cooked liver and ½-lb. kidneys a week. | 7.5.27.<br>R.B.C. 2,800,000.<br>15.5.27.<br>Feels much better. Spleen smaller.<br>28.5.27.<br>Feels very well.<br>R.B.C. 3,950,000.<br>Hb. 80 per cent.<br>C.I. 1·0.<br>Still slight variation in size and shape.<br>Discharged on Arsenic and HC1.<br>October, 1927.<br>Relapsed.<br>February, 1928.<br>Good response.<br>R.B.C. 3,000,000.<br>Good appetite.<br>April, 1931.<br>Feels very well. At one time tried liver extract, but found she was better with fresh liver. |

| Name.<br>Age.<br>Admitted. | Complaint. | History of present illness. | General condition. | Alimentary system. | C.N.S. | Blood. | Treatment. | Progress. |
|---|---|---|---|---|---|---|---|---|
| G.B.<br>46 years.<br>2.5.27. | Great weakness.<br>Vomiting. | Started a year ago ; been worse since confinement six months ago. | Typical lemon colour. | Teeth very bad.<br>Spleen, not felt. | Knee-jerks nil.<br>Plantar reflex flexor.<br>Sensation poor in both legs. | R.B.C. 920,000.<br>Hb. 25 per cent.<br>C.I. 1·25.<br>W.B.C. 6,900.<br>Poikilocytosis, anisocytosis.<br>Nucleated reds. | Liq. Ferri. Perchlor, m.7.<br>Liq. Arsen.<br>Liq. Strych.<br>Hyd. m. 3 t.d.s.<br>HCl dil. m. 30.<br>19.5.27.<br>Off arsenic.<br>30.5.27.<br>On arsenic again. | 15.5.27.<br>No better.<br>19.5.27.<br>Diarrhœa.<br>R.B.C. 1,000,000.<br>30.5.27.<br>Improving.<br>R.B.C. 1,250,000.<br>7.6.26.<br>7.6.27.<br>Went home.<br>April, 1931.<br>Not heard of again. |
| A.E.<br>54 years.<br>30.4.28. | Weakness and paleness. | Started eighteen months ago.<br>Vomits much. | Very anæmic. | Tongue, raw and red.<br>Teeth, poor.<br>Spleen, not felt. | Normal. | R.B.C. 1,800,000.<br>Hb. 5 per cent.<br>Poikilocytosis, anisocytosis.<br>Nucleated reds. | Liver extract ʒii t.d.s.<br>HCl dil. m. 30.<br>Liq. Arsenicalis m. 4 t.d.s.<br>Teeth extracted. | 8.5.28.<br>R.B.C. 2,600,000.<br>Hb. 60 per cent.<br>18.5.28.<br>R.B.C. 3,400,000.<br>Hb. 70 per cent.<br>1.6.28.<br>R.B.C. 3,900,000.<br>Hb. 20 per cent.<br>12.6.28.<br>R.B.C. 4,100,000.<br>Hb. 70 per cent.<br>4.7.28.<br>Feels very well.<br>R.B.C. 4,200,000.<br>Hb. 75 per cent.<br>Discharged. To continue treatment at home.<br>May, 1931.<br>Still well ; taking liver and liver extract. |

| | | | | | | | | |
|---|---|---|---|---|---|---|---|---|
| O.C.<br>49 years.<br>17.4.30. | *Anæmia.* | *Feeling weak*<br>*for last six*<br>months.<br>Appetite poor.<br>Numbness<br>and tingling<br>in legs. | *Thin and*<br>pale. | *Teeth, fair.*<br>No free HCl.<br>Spleen, not felt. | *Poor*<br>vibration<br>sense,<br>otherwise<br>normal. | *R.B.C. 2,400,000.*<br>Hb. 55 per cent.<br>C.I. 1·1.<br>Variation in size<br>and shape.<br>Several poorly<br>staining cells.<br>No nucleated red<br>cells seen. | ½-lb. *lightly*<br>cooked liver<br>daily.<br><br>3.6.30.<br>To take liver<br>extract ʒi<br>t.d.s. and<br>HCl m. 30<br>t.d.s. | 7.5.30.<br>R.B.C. 3,000,000.<br>Hb. 65 per cent.<br>18.5.30.<br>Hb. 65 per cent.<br>27.5.30.<br>Has severe cold and<br>is not so well.<br>R.B.C. 4,300,000.<br>Hb. 65 per cent.<br>3.6.30.<br>Much better.<br>Discharged.<br>May, 1931.<br>Still on liver, but<br>given up extract.<br>HCl. Feels fairly<br>well, but gets giddy<br>at times.<br>Occasionally gets<br>tingling in legs.<br>R.B.C. 4,100,000.<br>Hb. 70 per cent.<br>Anisocytosis.<br>No nucleated reds.<br>B.P. $\frac{164}{110}$<br>C.N.S. normal.<br>February, 1932.<br>Feels very well, and<br>still on liver extract. |

| Name.<br>Age.<br>Admitted. | Complaint. | History of present illness. | General condition. | Alimentary system. | C.N.S. | Blood. | Treatment. | Progress. |
|---|---|---|---|---|---|---|---|---|
| A.C.<br>62 years.<br>11.10.30. | Anæmia and dyspepsia. | Been getting bad for two years, but much worse last two months. | Very thin and anæmic. | Teeth, dentures.<br>Spleen enlarged.<br>No free HCl. | Normal. | R.B.C. 1,000,000.<br>Hb. 23 per cent.<br>C.I. 1·1.<br>Much poikilocytosis anisocytosis and nucleation of red cells. | 18.10.30.<br>Liver 6 oz. and liver extract on alternate days.<br>1.11.30.<br>Is having liver every day instead of extract. | 18.10.30.<br>Weaker and lost 4 lbs. in weight.<br>R.B.C. 900,000.<br>25.10.30.<br>R.B.C. 1,250,000.<br>Hb. 30 per cent.<br>Reticulocytes 15 per cent.<br>1.11.20.<br>R.B.C. 2,000,000.<br>Hb. 40 per cent.<br>Reticulocytes 12 per cent.<br>8.11.30.<br>General condition very much better.<br>R.B.C. 3,000,000.<br>Hb. 65 per cent.<br>23.11.30.<br>Has gained 15 lbs. since admission.<br>R.B.C. 4,200,000.<br>Hb. 75 per cent.<br>Still some poikilocytosis and anisocytosis.<br>No reticulocytes seen.<br>Discharged. |

| | | | | | | | | |
|---|---|---|---|---|---|---|---|---|
| *L.P.*<br>*65 years.*<br>*9.11.30.* | *Weakness, insomnia, frequency of micturition.* | *Getting bad for six months.* | *Very pale and thin.* | *Teeth, dentures. Spleen, not felt.* | *Marked mental changes.* Knee-jerks absent. Plantar reflexes extensor. Incontinence of urine and fæces. Sensation poor in all limbs. | *R.B.C. 1,260,000. Hb. 25 per cent.* Much variation in size and shape. Several megaloblasts. | *Cooked liver but cannot take it.*<br>11.11.30.<br>Liver extract ʒi t.d.s. | *11.11.30.*<br>*Much vomiting.*<br>13.11.30.<br>Unconscious all day.<br>14.11.30.<br>Died.<br>P.M. Typical changes of pernicious anæmia. |
| L.E.<br>55 years.<br>8.1.31. | Loss of use of legs. | Numbness in toes started two years ago and gradually lost power of legs. Appetite poor. Much vomiting. Always been anæmic. | Thin and pale. One breast removed fifteen years ago. | Tongue, smooth and red. Spleen, not felt. No free HCl. | Knee-jerks, absent. Plantar reflexes flexor. Sensation poor. | R.B.C. 1,240,000. Hb. 50 per cent. Poikilocytosis, anisocytosis, megaloblasts. | Liver extract ʒi t.d.s. | 11.1.31.<br>Cannot pass water. Delirious. R.B.C. 1,200,000. Hb. 40 per cent.<br>18.1.31.<br>Died.<br>P.M. Changes of pernicious anæmia also cystitis and pyelonephritis. |

| Name.<br>Age.<br>Admitted. | Complaint. | History of present illness. | General condition. | Alimentary system. | C.N.S. | Blood. | Treatment. | Progress. |
|---|---|---|---|---|---|---|---|---|
| T.C.<br>38 years.<br>21.1.31. | Numbness in hands and feet for four months.<br>Palpitations.<br>Sore tongue, getting yellow. | — | Yellow colour. | Teeth, dentures.<br>Tongue, pale and smooth.<br>Spleen, not felt.<br>No free HCl. | All reflexes brisk, otherwise normal. | R.B.C. 2,600,000.<br>Hb. 70 per cent.<br>Poikilocytosis, anisocytosis, polychromasia, punctate basophylia megaloblasts.<br>V.D.B. indirect 4 units. | 11.2.31.<br>Cooked liver and kidneys.<br>Liq. Arsenicalis m. 8, Ac. HCl dil. m. 45 t.d.s.<br><br>28.2.31.<br>½-lb. liver a day only.<br><br>May, 1931.<br>Is having liver extract, cooked liver and HCl. | 11.2.31.<br>Condition about the same.<br><br>20.2.31.<br>Improved.<br>R.B.C. 2,900,000.<br>Hb. 75 per cent.<br><br>28.2.31.<br>R.B.C. 3,300,000.<br>Hb. 75 per cent.<br>Jaundice gone.<br><br>14.3.31.<br>R.B.C. 4,200,000.<br>Hb. 80 per cent.<br>Slight variation in size and shape of red cells. numbness disappeared.<br>Discharged.<br><br>May, 1931.<br>Still very well.<br>R.B.C. 4,500,000.<br>Hb. 80 per cent. |

| | | | | | | | | |
|---|---|---|---|---|---|---|---|---|
| B.S.<br>56 years.<br>17.7.30. | General weakness. | Very well until three months ago, when he had pains over heart and tingling in left hand. Indigestion. | Lemon colour. Systolic murmur over whole of præcordium | Teeth, false. Tongue, furred. Spleen, just felt. No free HCl. | Subjective sensations only. | R.B.C. 1,100,000. Hb. 45 per cent. Poikilocytosis, anisocytosis, punctate basophylia, nucleated red cells. V.D.B. 1 unit indirect. | Ventriculin 10 gms. b.d. (1 gm. Ventriculin =12 gms. whole stomach.)<br><br>26.9.30.<br>Ventriculin 10 gms. daily. | 24.7.30.<br>Improved generally. R.B.C. 1,600,000.<br><br>3.8.30.<br>No indigestion. R.B.C. 2,700,000. Hb. 50 per cent.<br><br>19.8.30.<br>R.B.C. 3,100,000. Hb. 60 per cent.<br><br>3.9.30.<br>Feels very well. R.B.C. 4,000,000. Hb. 65 per cent. Variation in size and shape, but no nucleated red cells. Discharged. To continue with 10 gms. Ventriculin a day.<br><br>November, 1930.<br>Died. Had not been taking Ventriculin regularly. |

| Name.<br>Age.<br>Admitted. | Complaint. | History of present illness. | General condition. | Alimentary system. | C.N.S. | Blood. | Treatment. | Progress. |
|---|---|---|---|---|---|---|---|---|
| J.T.<br>50 years.<br>29.9.30. | Sent in by a cottage hospital for spleno-megaly. | Aching pain in left side for six months.<br>Appetite poor.<br>Vomiting and diarrhœa.<br>Tingling in legs and too weak to walk for the last three weeks.<br>Sore tongue. | Consider-ably jaundiced.<br>Mucous membranes pale. | Teeth, very poor.<br>Tongue, marked superficial glossitis.<br>No free HCl.<br>Spleen, reached nearly to the umbilicus. | Knee-jerks, left absent,<br>Plantar reflex, left extensor.<br>Poor sensation in both legs. | R.B.C. 1,900,000.<br>Hb. 50 per cent.<br>C.I. 1·3.<br>Marked poikilocytosis, anisocytosis with megaloblasts, punctate basophilia.<br>V.D.B. 7·5 units indirect. | Ventriculin gm. 10 b.d.<br><br>Teeth extracted two by two. | 4.10.30.<br>Feels better and appetite has improved.<br>R.B.C. 2,000,000.<br>Hb. 50 per cent.<br>Reticulocytes 5 per cent.<br>10.10.30.<br>Diarrhœa has stopped.<br>R.B.C. 2,800,000.<br>Hb. 55 per cent.<br>Reticulocytes 18 per cent.<br>17.10.30.<br>Jaundice practically gone.<br>Tongue quite better.<br>R.B.C. 3,900,000.<br>Hb. 65 per cent.<br>Reticulocytes 6 per cent.<br>25.10.30.<br>General condition very much better.<br>Has a very high colour.<br>R.B.C. 4,900,000.<br>Hb. 90 per cent.<br>Reticulocytes 2 per cent. |

| | | | | | | | | |
|---|---|---|---|---|---|---|---|---|
| | | | | | | | | *1.11.30.* *Still well, but still slight pins and needles in feet. Reflexes as before. Spleen much smaller. R.B.C. 5,000,000. Hb. 90 per cent.* |
| | | | | | | | 8.11.30. Ventriculin 10 gms. a day. | 8.11.30. R.B.C. 5,200,000. Hb. 90 per cent. 20.11.30. Still well. 1.12.30. R.B.C. 5,100,000. Hb. 85 per cent. Spleen cannot be felt. Still tingling in feet. |
| | | | | | | | 20.12.30. Ventriculin 10 gms. b.d. | 20.12.30. Not so well. R.B.C. 3,600,000. Hb. 55 per cent. 5.1.31. Improving. 10.1.31. R.B.C. 4,000,000. Hb. 75 per cent. 20.1.31. Feels quite well again R.B.C. 5,300,000. Hb. 100 per cent. Spleen not felt ; still tingling in legs ; reflexes as before. Discharged. April, 1931. Feels very well, but still has tingling in feet. Has given up Ventriculin for last month and been having 3 lbs. liver a week. |

| Name. Age, Admitted. | Complaint. | History of present illness. | General condition. | Alimentary system. | C.N.S. | Blood. | Treatment. | Progress. |
|---|---|---|---|---|---|---|---|---|
| E.N.<br>48 years.<br>15.12.30. | Tingling in arms and legs.<br>Anæmia. | Has been feeling run down for a year. Tingling for three months.<br>Appetite very poor. Much diarrhœa. | Rather pale. | Teeth, dentures.<br>Spleen, not felt.<br>No free HCl. | Much diminished sensation in all limbs.<br>Reflexes normal. | R.B.C. 2,600,000.<br>Hb. 65 per cent.<br>Poikilocytosis, anisocytosis, megaloblasts. | Ventriculin gms. 10 b.d.<br>22.12.30.<br>Ac HCl. dil. m 30 t.d.s. added.<br>20.1.31.<br>Going to have ½-lb. liver six days a week together with HCl and iron. | 22.12.30.<br>Appetite still very bad and much vomiting.<br>27.12.30.<br>Feels much better and can eat. Still tingling.<br>R.B.C. 3,000,000.<br>Reticulocytes 4 per cent.<br>1.1.31.<br>R.B.C. 3,200,000.<br>Hb. 70 per cent.<br>Reticulocytes 4 per cent.<br>8.1.31.<br>Very much better and eats well.<br>R.B.C. 4,000,000.<br>No reticulocytes seen<br>20.1.31.<br>Still much improving and tingling less.<br>R.B.C. 4,500,000.<br>Hb. 80 per cent.<br>Slight variation in size and shape. No nucleated red cells.<br>Discharged.<br>April, 1931.<br>Still very well. |

| | | | | | | | | |
|---|---|---|---|---|---|---|---|---|
| *E.F.*<br>*37 years.*<br>*23.1.31.* | *Inability to walk, with tingling in arms and legs.*<br>Shortness of breath.<br>Flatulence. | *Started three months ago.* | *Rather anæmic and thin.* | *Teeth, dentures.*<br>*Spleen, not felt.* | *Sensation diminished in forearms and legs.*<br>Knee-jerks, both increased.<br>Left extensor, plantar reflex.<br>No clonus.<br>Abdominal reflex, absent. | *R.B.C. 2,720,000.*<br>*Hb. 50 per cent.*<br>*C.I. 0·95.*<br>*Poikilocytosis, anisocytosis.*<br>*Nucleated red cells.* | *Liver 6 oz. daily.*<br>*HCl. m. 20 t.d.s.*<br>*3.2.31.*<br>Liver extract ʒii t.d.s. instead of liver.<br>20.2.31.<br>Off liver extract and put on Ventriculin 10 gms. b.d.<br>22.3.31.<br>Extomak 2 phials a day instead of Ventriculin. | *3.2.31.*<br>*Condition about the same; can't take the liver.*<br>*10.2.31.*<br>Slight general improvement, but very poor appetite.<br>R.B.C. 2,900,000.<br>Hb. 50 per cent.<br>Reticulocytes 2 per cent.<br>20.2.31.<br>Still about the same. Much troubled by tingling<br>24.2.31.<br>Feels definitely better<br>28.2.31.<br>Colour much better. Tingling lessened. Appetite improved.<br>R.B.C. 3,270,000.<br>Hb. 50 per cent.<br>Reticulocytes 2½ per cent.<br>14.3.31.<br>A good deal better.<br>R.B.C. 3,800,000.<br>Hb. 60 per cent.<br>22.3.31.<br>Still improving.<br>4.4.31.<br>Very much better. Tingling nearly gone. Reflexes as before.<br>R.B.C. 4,570,000.<br>Hb. 65 per cent.<br>Slight variation in size and shape.<br>28.4.31.<br>Eating very well; still slight tingling. Discharged. To continue same treatment |

| Name. Age. Admitted. | Complaint. | History of present illness. | General condition. | Alimentary system. | C.N.S. | Blood. | Treatment. | Progress. |
|---|---|---|---|---|---|---|---|---|
| E.C.<br>51 years.<br>18.3.31. | Shortness of breath and tiredness. | — | Very anæmic. | Teeth, false.<br>No glossitis.<br>Spleen, not felt.<br>No free HCl. | Subjective sensations only. | R.B.C. 2,520,000.<br>Hb. 60 per cent.<br>Poikilocytosis, anisocytosis.<br>No nucleated red cells seen. | Extomak 2 phials daily. | 26.3.31.<br>Feels much better.<br>R.B.C. 3,300,000.<br>Hb. 65 per cent.<br>1.4.31.<br>R.B.C. 3,600,000.<br>10.4.31.<br>R.B.C. 4,800,000.<br>Hb. 75 per cent.<br>18.4.31.<br>R.B.C. 5,260,000.<br>Hb. 85 per cent.<br>Still slight poikilocytosis and anisocytosis.<br>General condition very good.<br>Discharged.<br>May, 1931.<br>Feels and looks well.<br>Been having ½-lb. lightly cooked liver a day.<br>R.B.C. 4,900,000. |
| M.G.<br>35 years.<br>30.1.31. | Has been getting very weak for the last year, since confinement.<br>Diarrhœa.<br>Vomiting. | — | — | Tongue, normal.<br>Teeth, false.<br>Spleen, not felt. | Knee-jerks brisk.<br>No clonus.<br>Plantar reflex doubtful. | R.B.C. 1,900,000.<br>Hb. 45 per cent.<br>C.I. 1·1.<br>Anisocytosis, poikilocytosis, polychromasia.<br>Nucleated cells. | ½-lb. raw hog's stomach daily, HCl. m. 30 t.d.s.<br>5.2.31.<br>Gastrex instead of raw stomach. | 10.2.31.<br>Decided improvement all round.<br>R.B.C. 2,125,000.<br>Hb. 45 per cent.<br>Reticulocytes 5 per cent.<br>17.2.31.<br>R.B.C. 2,950,000.<br>Hb. 50 per cent.<br>Reticulocytes 4 per cent.<br>3.3.31.<br>Very much better and no bad symptoms.<br>R.B.C. 4,500,000.<br>Hb. 55 per cent.<br>Discharged. |

| | | | | | | | | |
|---|---|---|---|---|---|---|---|---|
| | | | | | | | | *May, 1931.*<br>*Been continuing with stomach preparation since discharge.*<br>*Well up to a fortnight ago, but since then has not felt at all well, but not as bad as before going to hospital. Gets pins and needles in arms and legs. Tongue sore. Anæmic and jaundiced. R.B.C. 2,100,000. Hb. 40 per cent.* |
| F.S.<br>52 years.<br>26.1.31. | General weakness. | No energy for four months. Appetite bad. Pneumonia four years ago, and never been the same since. | Pale lemon colour. Teeth, dentures. | Superficial glossitis. Spleen, enlarged. No free HCl. | No change. | R.B.C. 1,980,000. Hb. 35 per cent. C.I. 1·0. Poikilocytosis, anisocytosis, polychromasia. Punctate basophilia, megaloblasts. | Liver extract ʒii t.d.s. HCl. | 5.2.31.<br>Appetite better.<br>9.2.31.<br>R.B.C. 2,900,000. Hb. 45 per cent.<br>20.2.31.<br>R.B.C. 3,930,000. Hb. 60 per cent. Anisocytosis, poikilocytosis. Jaundice gone. Tongue better.<br>5.3.31.<br>Feels very well. R.B.C. 5,200,000. Hb. 70 per cent. Discharged.<br>May, 1931.<br>Has been on same treatment since discharge. Does not feel as well as a month ago and easily gets tired. R.B.C. 3,500,000. Hb. 60 per cent. |

*Summary and Conclusions :—*

*Arsenic.*—Up till four or five years ago this was by far the best method of treating the disease. Even with its use the average duration of life from the start of symptoms was only six months to two years, and only two cases of cure have been reported on it. With its use during the best remissions the red corpuscles rarely rose higher than 3,000,000 per c.mm.

*Iron.*—This is of no use by itself, but is useful in conjunction with other treatments ; it is a producer of hæmoglobin rather than a stimulating agent to the formation of red-blood corpuscles.

*Operative Methods.*—The only two procedures which are of use are (*a*) transfusion and (*b*) removal of easily accessible septic foci. The former is only useful in tiding over a critical time, and for the latter the removal of septic teeth is a great adjunct to treatment. In Percy's procedure of removing the gall-bladder, appendix and spleen it appears to have been the splenectomy which was the important part, as those patients who did not have this organ removed did not do as well as those who did. On the whole, though, splenectomy is not worth performing.

*Hydrochloric Acid.* — This will not cure the achlorhydria, but in small doses is sometimes useful as a gastric stimulant, and thus has an indirect therapeutic use by enabling the patients to take their liver or stomach foods more readily.

*Liver.*—This was an immense advance on anything which had as yet been tried in the treatment of pernicious anæmia. The following are the chief points in its use :—

(*a*) From half to a quarter of a pound at least should be taken daily.

(*b*) It has its maximum effect when taken raw, but may be lightly cooked in any way desired.

(*c*) An increase in the reticulocytes occurs. Normally this is from $\frac{1}{2}$ to 2 per cent., but during liver treatment they rise to 10 to 15 per cent., and are at their maximum about the end of the first week, and then gradually fall, reaching their normal number during the third week.

(*d*) The lower the initial red-blood count the higher is the reticulocyte peak. A low reticulocyte count with a low initial red count indicates a bad prognosis or insufficient liver.

(*e*) On full doses of liver the red-blood corpuscles eventually reach 4 to $4\frac{1}{2}$ million, and occasionally over 5 millions.

(*f*) The patients' colour is no indication of their true red-blood count, which is often lower than their appearance suggests.

(*g*) An improvement in the appetite, a pink flush over the malar bones and subsidence of the diarrhœa are often the first signs of improvement, and occur usually between the fourth and seventh day.

(*h*) An increase in the red-blood cells is apparent on the fifth or sixth day, and is preceded by increased reticulocytes.

(*i*) The soreness of the tongue is soon diminished.

(*j*) The jaundice rapidly fades, and the Van den Bergh test is usually normal in a fortnight.

(*k*) The nervous symptoms are the last to improve. Only the subjective signs usually improve, but definite organic changes remain the same, although two cases of a reversion to a flexor plantar reflex have been recorded at Newcastle.

(*l*) Hydrochloric acid makes no difference to the majority of patients, but is beneficial to some.

(*m*) Free hydrochloric acid practically never returns to the stomach.

(*n*) Any superimposed infection, however slight, diminishes the effectiveness of the treatment.

(*o*) Although the patient may at first dislike liver, he often gets a craving for it when he has started to improve.

(*p*) The larger the amount taken the more rapid is the reticulocyte response. Occasional massive doses may be given instead of repeated small ones; it is the total amount of liver taken that matters, not the daily dose.

(*q*) The discovery of the effective fraction of the liver was a great advance, as it is easy to take, whereas many people cannot take whole liver.

(*r*) When liver extract is being used it has to be given in slightly larger doses than its equivalent of whole liver.

(*s*) It has been found in several cases that previous transfusion detracts from the efficiency of liver.

(*t*) There have been a very few instances of gout and venous thrombosis ensuing after liver treatment, but these can be eliminated by using the extract.

(*u*) In the years 1921–26 the mortality from pernicious anæmia was unchanged; in 1927 and still more in 1928 there was a definite reduction in mortality from pernicious anæmia, there being 915 less deaths from this disease than was expected. The improvement was greatest in London and least in the small urban districts. All this is what one would have expected from an increasing use of a new beneficial treatment.

(*v*) It has been said by many that if there is no improvement on liver either not enough liver is being taken or the case is not one of pernicious anæmia.

*Hog's Stomach.*—Up to the present time by far the best results have been achieved by the use of this. By the regular use of this it may be said that pernicious anæmia is a definitely curable disease. Whereas the use of liver merely supplies the patient with the ready-made substance that is essential for the formation of the red cells, the use of stomach tissue supplies them with that which is wanted to enable them to make their own red-blood cell formative material.

(*a*) The remissions that take place after the use of this are of the same nature as those caused by liver, but occur more rapidly. Reticulocyte crises after stomach tissue often reach from 20 to 40 per cent. when the substance is given in sufficient doses; at Bristol, though, the highest recorded is only 16 per cent., but in no case where stomach has been used has the initial red count been below 1,500,000, and more than equivalent of 240 gms. a day have been used.

(*b*) The addition of hydrochloric acid is much more rarely needed when stomach is used than when liver is the treatment.

(*c*) Very few deaths have been recorded in those taking the treatment, and in nearly every case it was found that the stomach extract was not being taken regularly. Wilkinson reports that of 108 cases 92·7 per cent. after eighteen months are quite free from symptoms, and a further 6 per cent. have nerve trouble but are otherwise well.

(*d*) Previous blood transfusions appear to make no difference to the result.

(*e*) Several cases which have failed to respond to liver in adequate amounts have immediately improved on starting to take hog's stomach, either raw or as a desiccated preparation.

(*f*) If it can only doubtfully be said that absence of improvement with liver indicates that the disease is not pernicious anæmia, it can certainly be said so of hog's stomach.

REFERENCES.

Anderson, J. H., and Spriggs, E. J., *Lancet*, 1927, ii. 958.

Bowen, B. D., *Jour. Amer. Med. Assoc.*, 1930, xcv. 30.

Bramwell, B., *Med. Times and Gazette*, 1877, ii. 323, 429.

Conner, H. M., *Jour. Amer. Med. Assoc.*, 1930, xciv. 388.

East, C. F. T., *Brit. Med. Jour.*, 1928, i. 491.

Fleming, G. W. T. H., *Brit. Med. Jour.*, 1929, i. 638.

Fraser, T. R., *Brit. Med. Jour.*, 1894, i. 1,172.

Holmes, A. H., *Brit. Med. Jour.*, 1929, ii. 12.

Hunter, W., *Brit. Med. Jour.*, 1890, ii. 1, 81.

Isaacs, R., and Sturges, C. C., *Jour. Amer. Med. Assoc.*, 1930, xcv. 585 ; *ibid.*, 1929, xciii. 747 ; *Amer. Jour. Med. Sci.*, 1930, p. 180.

Knott, F. A., *Lancet*, 1928, ii. 968.

McKinlay, P. L., *Lancet*, 1929, ii. 1,086.

Medical Research Council, *Brit. Med. Jour.*, 1928, i. 398, 463.

Minot, G. R., and Murphy, W. P., *Jour. Amer. Med. Assoc.*, 1926, lxxxvii. 470 ; *ibid.*, 1927, lxxxix. 759 ; *Brit. Med. Jour.*, 1927, ii. 674.

Murphy, W. P., and Munroe, R. T., *Jour. Amer. Med. Assoc.*, 1927, lxxxviii. 1,211.

Ordway, T., and Gorham, L. W., *Jour. Amer. Med. Assoc.*, 1928, xci. 925.

Panton, P. M., and Maitland Jones, A. G., *Lancet*, 1923, i. 274.

Percy, N. M., *Trans. Amer. Surg. Assoc.*, 1920, xxxviii. 451.

Phillips, F. A., *Brit. Med. Jour.*, 1928, i. 93.

Regnikoff, P., *Jour. Amer. Med. Assoc.*, 1929, xciii. 367.

Roy. Soc. Med., *Lancet*, 1931, i. 702.

Spence, J. C., *Lancet*, 1927, ii. 1,026.

Stockman, R., *Brit. Med. Jour.*, 1895, i. 965, 1,025, 1,083.

Ungley, C. C., and Suzman, M. M., *Brain*, 1929, lii. 271.

Whipple, G. H., and Robbins, F. S., *Amer. Jour. Physiol.*, 1925, lxxii. 395 ; *Jour. Amer. Med. Assoc.*, 1928, xci. 863.

Wilkinson, J. F., and Brockbank, W., *Brit. Med. Jour.*, 1929, ii. 12 ; *ibid.*, 1930, i. 236 ; *ibid.*, 1931, i. 85.

